# Syncope as Initial Presentation in an Undifferentiated Type Acute Myeloid Leukemia Patient with Acute Intracranial Hemorrhage

**DOI:** 10.3390/brainsci9080207

**Published:** 2019-08-20

**Authors:** Meng-Yu Wu, Ching-Hsiang Lin, Yueh-Tseng Hou, Po-Chen Lin, Giou-Teng Yiang, Yueh-Cheng Tien, Hsiao-Ching Yeh

**Affiliations:** 1 Department of Emergency Medicine, Taipei Tzu Chi Hospital, Buddhist Tzu Chi Medical Foundation, New Taipei 231, Taiwan; 2 Department of Emergency Medicine, School of Medicine, Tzu Chi University, Hualien 970, Taiwan; 3 Psychiatry Department, Chang Bing Show-Chwan Memorial Hospital, Changhua 505, Taiwan

**Keywords:** syncope, acute myeloid leukemia, intracranial hemorrhage, hyperleukocytosis, blast crisis

## Abstract

Intracranial hemorrhage (ICH) is a catastrophic complication in patients with acute myeloid leukemia (AML). AML cells, especially in the acute promyelocytic leukemia subtype, may release microparticles (MPs), tissue factor (TF), and cancer procoagulant (CP) to promote coagulopathy. Hyperfibrinolysis is also triggered via release of annexin II, t-PA, u-PA, and u-PAR. Various inflammatory cytokines from cancer cells, such as IL-1β and TNF-α, activate endothelial cells and promote leukostasis. This condition may increase the ICH risk and lead to poor clinical outcomes. Here, we present a case under a unique situation with acute ICH detected prior to the diagnosis of AML. The patient initially presented with two episodes of syncope. Rapidly progressive ICH was noted in follow-up computed tomography (CT) scans. Therefore, we highlight that AML should be among the differential diagnoses of the etiologies of ICH. Early diagnosis and timely intervention are very important for AML patients.

## 1. Introduction

Intracranial hemorrhage (ICH) is an uncommon complication in patients with acute myeloid leukemia (AML), and coincides with poor clinical outcome. The mortality rate of AML patients developing acute ICH is high and death can occur within days if not diagnosed and treated appropriately. In a study by Owattanapanich et al. [1], 4.29% (38/685 patients) AML patients presented with ICH and acute promyelocytic leukemia had a higher risk of ICH with odds ratio of 6.15, compared to other types in the AML subgroup analysis [1]. Few patients with acute myeloid leukemia in undifferentiated type presented syncope and ICH as an initial presentation. AML with undifferentiated type (AML-M1) patients were presented with ICH accounting for only 15.79% (6/38 patients). In addition, only 10% of patients present hyperleukocytosis, which are at higher risk of tumor lysis syndrome and leukostasis [2]. Several risk factors were investigated, including hypertension, vasculopathy, thrombocytopenia, lower coagulation factors, disseminated intravascular coagulation (DIC), and hyperleukocytosis. Hyperleukocytosis and DIC were two major factors associated with ICH. The incidence of hyperleukocytosis in acute AML patients was 5–20% and this condition may be significantly associated with DIC, leukostasis, and tumor lysis syndrome, promoting ICH events. However, clinical experiences concerning ICH in AML are limited in the literature. Therefore, early diagnosis and timely intervention are crucial for AML patients. Here, we describe a rare case of syncope at initial presentation in an AML-M0 patient with acute intracranial hemorrhage.

## 2. Case Presentation

This case report was approved by the Institutional Review Board of Taipei Tzu Chi Hospital, Buddhist Tzu Chi Medical Foundation (IRB number: 08-CR-060).

**Figure 1:**
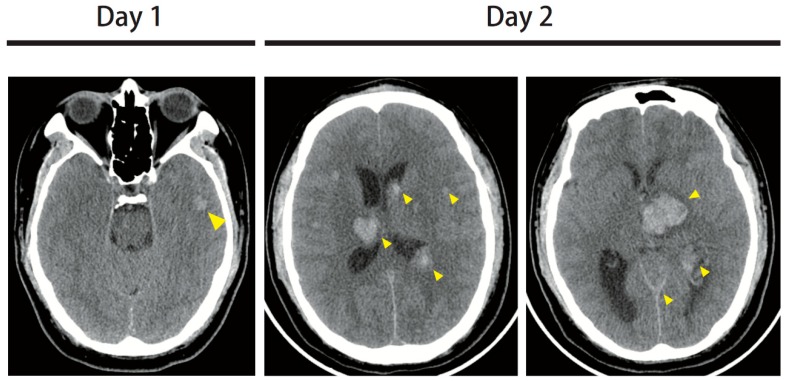
On the day 1 brain computed tomography (CT), a 13 mm lesion with hyperdensity was found in the left temporal region, with suspected intracerebral hemorrhage. On the day 2 brain CT, multifocal intracranial hemorrhages in bilateral cerebral hemispheres were noted, the largest being a 30 mm lesion in the left thalamus. The midline structures were shifted to the right side. Extensive swelling was present in the cerebellum and brain stem.

A 31-year-old male presented with a productive cough and rhinorrhea for 4 days. He had no past medical history or medicine history. The symptoms were associated with intermittent high fever up to 39–40 °C and myalgia. He took symptomatic treatment but in vain. The patient went to a local medical doctor for treatment. While waiting outside the consulting room, syncope occurred twice and he contused the frontal area. There was no tonic or clonic seizure. Other symptoms, including skin rash, arthritis, or tendency for abnormal bleeding, were not found. Pneumonia-induced sepsis was suspected, and he was transferred to our emergency department. Upon admission, his temperature was 38.4 °C, blood pressure was 110/62 mmHg, heart rate was 129 beats/min, body weight was 90.6 kg, and height was 173 cm. Upon physical examination, his Glasgow Coma Score (GCS) score was E4V5M6 and bilateral pupil size was 2 mm with light reflex. There was no horizontal or vertical nystagmus. The neck was supple with no limited range of motion. The bilateral breath sound was clear without wheezing or crackle, and tachycardia was noted. There was no Babinski sign, decreased muscle power, or unsteady gait. The chest *X*-ray revealed no significant pulmonary nodules or pneumonia patch. An influenza A + B rapid screening test was performed, which showed negative results. A brain CT revealed a 13 mm lesion with hyperdensity in the left temporal region, with suspected intracerebral hemorrhage (Figure 1). Laboratory evaluation of the patient revealed severe leukocytosis with blastemia (Table 1).

Results from the bone marrow biopsy report showed 90% cellularity. Blasts accounted for more than 90% of all nucleated cells. Hypercellular and monotonous bone marrow was noted with undifferentiated myeloblasts with prominent, convoluted nuclei, and agranular cytoplasm. There were significantly decreased erythroid and megakaryocyte lineages. The immunohistochemical profile was as follows: CD34(+), CD117(+), MPO(+), CD33(+), CD68(–), hemoglobin A(–), Factor VIII(–), CD19(–), CD3(–), TdT(–), and PAX5(–). The peroxidase and alpha naphthyl acetate esterase (ANAE) test was positive and chloroacetate esterase (CAE) test was negative. Acute myeloid leukemia was diagnosed. Broad-spectrum antibiotic and adequate hydration were administered. An antineoplastic agent, hydroxyurea (15 mg/kg/day), and emergency leukocytapheresis were used to control disease progression. Unfortunately, the patient became drowsy with asymmetric pupil size and no light reflex. The follow-up brain CT showed multifocal intracranial hemorrhage in the bilateral cerebral hemispheres with midline shift involving the brain stem (Figure 1). Progressive hypotension was noted even using a vasopressor agent. Finally, the patient expired due to uncontrolled hemodynamic shock on the fourth day.

## 3. Discussion

**Figure 2:**
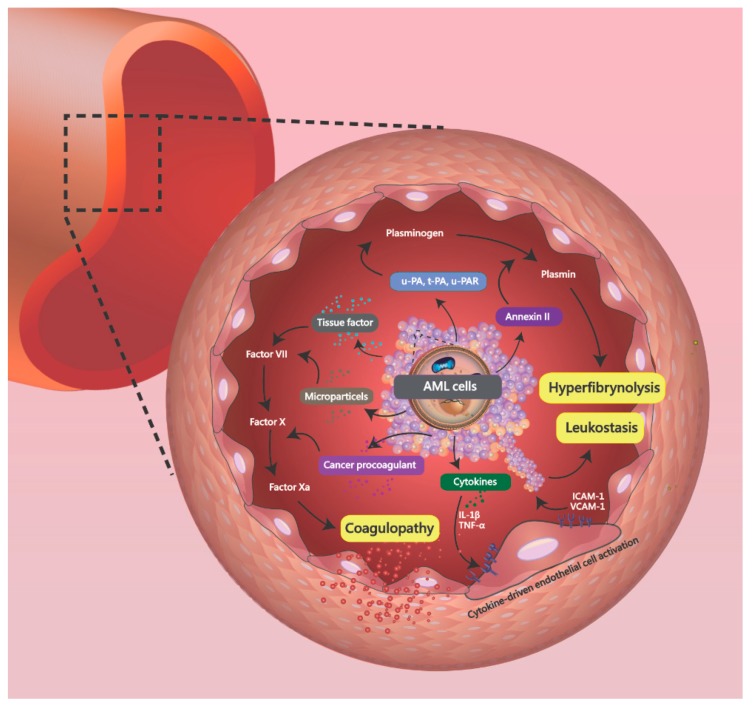
In an APL model, the acute myeloid leukemia (AML) cell produces microparticles (MPs), tissue factor (TF), and cancer procoagulant (CP), which act on the coagulation cascade to promote coagulopathy. The release of annexin II, t-PA, u-PA, and u-PAR from AML cells converts plasminogen into plasmin, causing hyperfibrinolysis. Various inflammatory cytokines from cancer cells, such as IL-1β and TNF-α, also activate endothelial cells and promote leukostasis.

In the AML population, intracranial-hemorrhage-induced syncope at initial presentation is an uncommon but fatal condition. The study by Balmages et al. [3] showed a similar AML case with hyperleukocytosis (WBC count of 51.7 × 10^9^/L) was reported. In those populations, there was a significantly higher risk of death from rapidly developed fatal ICH. In the study by Chen et al. [4], a total of 841 AML patients were enrolled, and 6% (51/841 patients) were diagnosed with ICH. The location of ICH was common at supratentorium (44/51 cases), followed by basal ganglion (9/51 cases), cerebellum (5/51 cases), and brainstem (4/51 cases). The analysis of clinical outcome revealed that 67% of patients (34 patients) died of ICH within 30 days of diagnosis. Severe DIC and leukostasis are two main causes leading to ICH. In the untreated AML population, 5–20% of patients may present with hyperleukocytosis, defined by white blood cell counts > 100,000/mL [5,6]. The hyperleukocytosis may result from a rapid blast proliferation and hematopoietic cell adhesion dysfunction [7]. Hyperleukocytosis may induce DIC, tumor lysis syndrome, and leukostasis. The brain and lungs are the common organs involved, and are associated with a high mortality rate. Severe hyperleukocytosis may induce mechanical obstruction of small vessels, causing malperfusion, endothelial damage, subsequent hemorrhage, and cell death [8]. The mechanical obstruction may be induced by myeloblasts via release of various inflammatory cytokines and factors, such as IL-1β and TNF-α, to promote endothelial cell activation [9]. The activated endothelial cells increase the expression of adhesion receptors, such as intracellular adhesion molecule-1 (ICAM-1) and vascular cell adhesion molecule-1 (VCAM-1) (Figure 2). However, cytokine-driven endothelial cell activation, which is induced by AML, may lead to a loss of vascular integrity and impaired endothelial antithrombotic function [10,11]. The endothelial damage can then lead to impaired myeloblast migration and subsequent hemorrhage.

In the study by Dixit et al. [12], DIC was found in 16.6% of AML patients (4/24 patients; M2: 1 case, M3: 2 cases, M5: 1 case). Acute promyelocytic leukemia is a subtype of AML, characterized by fatal bleeding events, and has also been well investigated [13]. Coagulopathy may be induced by AML cells via high expression of tissue factor, activating the coagulation cascade. In an APL model, the cells expressed high levels of three main procoagulants, including microparticles (MPs), tissue factor (TF), and cancer procoagulant (CP) [14]. TF is a cell surface receptor that catalyzes the conversion of factor X into factor Xa through factor VIIa. CP is a cysteine protease procoagulant involving the coagulation cascade by activating factor X to promote thrombin [15]. MPs decrease coagulation time in AML and promote coagulopathy via increased thrombin generation [16]. High levels of annexin II, t-PA, u-PA, and u-PAR were also found to activate plasminogen into plasmin in AML cells, which induced hyperfibrinolysis [17,18]. In addition, endothelial cells may be destroyed via release of various inflammatory cytokines, such as IL-1β and TNF-α, to promote DIC (Figure 2) [19].

Leukapheresis in AML patients with hyperleukocytosis rapidly removes excessive leukocytes by mechanical separation to control complications. One round of leukapheresis could reduce the WBC count by about 10–70% [20]. The efficacy of leukapheresis has been reported in previous clinical trials, but there are some trials that disagree. After leukapheresis, AML cells may rapidly mobilize from the bone marrow to peripheral blood. In addition, the benefit of leukapheresis was not significant in clinical trials and the long-term outcome was not changed. However, leukapheresis may be beneficial for preventing leukostasis and reducing the risk of ICH. A study by Novotny et al. [21] included 95 hyperleukocytic leukemia patients and evaluated the effectiveness of therapy using the four-stage clinical grading scale. The results showed that the AML M1/M2 population with hyperleukocytosis (*p* = 0.011), lower hemoglobin (*p* = 0.004), and blast crisis (*p* = 0.004) presented with highly probable leukostasis with a high score. A subgroup was given early leukapheresis treatment which showed a benefit (based on a grading score) in preventing leukostasis-related early death. In our case, hyperleukocytosis with thrombocytopenia and blast crisis was noted. Leukapheresis was performed but the treatment was not very effective. Persistent leukostasis and DIC promoted the progression of ICH with multiple focal hemorrhages in day 2 CT scans.

## 4. Conclusions

In this article, we present the rare and unusual case of a patient with AML-induced ICH initially presenting with syncope. In the AML population, DIC and leukostasis play a critical role in ICH. This detailed pathophysiology may improve physicians’ development of therapeutic strategies for AML-induced ICH. Here, we highlight the clinical features and etiologies of AML-induced ICH. Early diagnosis and timely leukapheresis may prevent fatal progressive complications.

## Figures and Tables

**Table 1:** The laboratory evaluation in this patient.

Variables	Normal Range	Patient Data			
		Day 1	Day 2	Day 3	Day 4
White cell count	3.5–11 × 10^9^/L	56.1	54.6	51.3	59.0
Band form neutrophils	0–3%	0.0%	0.0%	0.0%	0.0%
Segment form neutrophils	45–70%	0.0%	0.0%	0.0%	0.0%
Lymphocytes	25–40%	16.0%	7.0%	4.0%	6.0%
Eosinophils	1–3%	0.0%	0.0%	0.0%	0.0%
Monocytes	2–8%	6.0%	0.0%	0.0%	0.0%
Basophils	0–1%	0.0%	0.0%	0.0%	0.0%
Myelocytes	0.0%	0.0%	1.0%	0.0%	1.0%
Nucleated red blood cells	0.0%	0.0%	0.0%	0.0%	3.0%
Blast	0.0%	84.0%	92.0%	96.0%	93.0%
Hemoglobin	7.45–9.93 mmoL/L	5.40	4.65	4.47	5.28
Platelet counts	150–400 × 10^9^/L	64	54	146	130
Blood urine nitrogen	2.5–6.4 mmoL/L	7.5	7.9	7.1	----
Creatinine	0.04–0.09 mmoL/L	0.168	0.1591	0.1591	----
Sodium	136–145 mmoL/L	138	144	157	147
Potassium	3.5–5.1 mmoL/L	2.8	2.2	4.1	2.7
Glucose	3.9–5.6 mmoL/L	6.3	----	----	----
Alanine aminotransferase	0.27–1.05 µkat/L	0.50	----	0.55	----
High-sensitive Troponin I	0–19 ng/L	82.1	----	----	----
C-Reactive Protein	<31.4 nmoL/L	810.5	----	----	----
Prothrombin time	8.0–12.0 s	12.7	12.1	12.7	----
Partial thromboplastin time	23.9–35.5 s	26.8	26.5	25.0	----
FDP-Ddimer	0–500 µg/L	708.18	552.51	----	----
